# FT-NIR: a tool for rapid intracellular lipid quantification in oleaginous yeasts

**DOI:** 10.1186/s13068-019-1513-9

**Published:** 2019-06-29

**Authors:** Mikołaj Chmielarz, Sabine Sampels, Johanna Blomqvist, Jule Brandenburg, Frida Wende, Mats Sandgren, Volkmar Passoth

**Affiliations:** 1 Department of Molecular Sciences, Swedish University of Agricultural Sciences, Box 7015, 750 07 Uppsala, Sweden; 2 Faculty of Science and Technology, Norwegian University of Life Sciences: NMBU, P.O.Box 5003, 1432 Ås, Norway

**Keywords:** FT-NIR, Lipid quantification, *Rhodotorula toruloides*, *Lipomyces starkeyi*, *Yarrowia lipolytica*

## Abstract

**Background:**

Lipid extraction for quantification of fat content in oleaginous yeasts often requires strong acids and harmful organic solvents; it is laborious and time-consuming. Therefore, in most cases just endpoint measurements of lipid accumulation are performed and kinetics of intracellular lipid accumulation is difficult to follow. To address this, we created a prediction model using Fourier-transform near-infrared (FT-NIR) spectroscopy. This method allows to measure lipid content in yeast.

**Methods:**

The FT-NIR calibration sets were constructed from spectra of freeze-dried cells of the oleaginous yeasts *Rhodotorula toruloides* CBS 14, *Lipomyces starkeyi* CBS 1807 and *Yarrowia lipolytica* CBS 6114. The yeast cells were obtained from different cultivation conditions. Freeze-dried cell pellets were scanned using FT-NIR in the Multi Purpose Analyser (MPA) from Bruker. The obtained spectra were assigned corresponding to total fat content, obtained from lipid extraction using a modified Folch method. Quantification models using partial least squares (PLS) regression were built, and the calibration sets were validated on independently cultivated samples. The *R. toruloides* model was additionally tested on *Rhodotorula babjevae* DBVPG 8058 and *Rhodotorula glutinis* CBS 2387.

**Results:**

The *R*^2^ of the FT-NIR model for *R. toruloides* was 98%, and the root mean square error of cross-validation (RMSECV) was 1.53. The model was validated using a separate set of *R. toruloides* samples with a root mean square error of prediction (RMSEP) of 3.21. The *R*^2^ of the *Lipomyces* model was 96%, with RMSECV 2.4 and RMSEP 3.8. The *R*^2^ of the mixed model, including all tested yeast strains, was 90.5%, with RMSECV 2.76 and RMSEP 3.22, respectively. The models were verified by predicting the total fat content in newly cultivated and freeze-dried samples. Additionally, the kinetics of lipid accumulation of a culture were followed and compared with standard lipid extraction methods.

**Conclusions:**

Using FT-NIR spectroscopy, we have developed a faster, less laborious and non-destructive quantification of yeast intracellular lipid content compared to methods using lipid extraction.

## Background

Lipids from renewable sources are of particular interest due to their potential use as a sustainable feedstock for biodiesel and chemicals production, and as ingredients in food or animal feed [1–6]. Oleaginous yeasts can accumulate lipids to more than 20% of their dry weight. The basidiomycetous red yeast *Rhodotorula toruloides* has the ability to utilise and accumulate lipids using glycerol, glucose and xylose as sole carbon sources. Thus, *R. toruloides* has the potential to convert side products such as lignocellulose from forest and agricultural production systems, or crude glycerol from biodiesel transesterification, into lipids of higher value. There are also ascomycetous oleaginous yeasts of interest, e.g. *Lipomyces starkeyi* that is known for its high lipid content and potential for fuel production [2], and the nonpathogenic dimorphic aerobic yeast *Yarrowia lipolytica,* for its highly developed tool box for genetic modification, for instance, to yield high lipid content or the production of long-chain Ω-3 fatty acids [3].

One of the biggest challenges in optimising yeast-based lipid production systems is the ability to monitor lipid accumulation in yeast cells during their cultivation. Lipid extraction from microbes usually relies on organic solvents, often in the presence of strong acids and at elevated temperatures to break the cell walls [5–8]. Organic solvents are hazardous for the environment and to human health. Moreover, because extraction is performed in a biphasic system, errors can be introduced to lipid quantification due to pipetting errors. These errors can, however, be kept small by extracting lipids from large sample volumes. This can, however, affect experimental fermentations, since the total fermentation volume can be significantly changed during the time course of the cultivation by the removal of large sample volumes. Apart from this, lipid extraction is also labour-intensive and time-consuming. For these reasons, lipid production is in most cases monitored by endpoint measurement methods [7, 8], and the kinetics of lipid accumulation has rarely been investigated.

Infrared spectroscopy (IR) measures the absorption of light by fundamental molecular vibrations. It can be used to identify biomolecules such as proteins, lipids, DNA and other organic compounds [9]. Near-infrared (NIR) spectroscopy measures absorption of light vibrational overtones and their combinations. The NIR region of the electromagnetic wavelength spectrum ranges from 780 nm (wave number 12,800 cm^−1^) to about 2500 nm (4000 cm^−1^). At wavelengths above 2500 nm, the mid-IR region starts. Both IR and NIR spectroscopy have a number of useful applications, in industry and within research, for the identification and quantification of different organic compounds, product quality control and authentication [10–12]. These spectroscopic methods have also been applied for yeast species identification and strain differentiation [13, 14]. NIR spectroscopy among other methods is used to analyse sugar and lipid content in the bioethanol industry [15]. The absorption bands in NIR spectroscopy are broad and often overlapping, resulting in complex spectra that are difficult to interpret [16]. In classical NIR spectroscopy, the sample is scanned by a monochromatic light which is sequentially changed to different wavelengths over the whole NIR wavelength bandwidth. The principle of Fourier-transform (FT)-NIR is slightly different: a sample is exposed to polychromatic light, which is then divided at the beam splitter resulting in a so-called interferogram showing the intensity of the light as a function of time. The interferogram is then transformed to the frequency domain by Fourier transformation. The advantage of FT-NIR compared to classical NIR spectroscopy is that it is faster, as it allows simultaneous measurement over the whole wavelength range, is more sensitive as there is a lower background noise, and has a higher precision [16]. Both IR and NIR spectroscopy techniques have been used before, for example, to analyse lipids in the human body in a non-invasive way [17], lipid content and type in plants [18], to determine olive oil quality [19], or to analyse lipids in milk [20], yeasts [21, 22] and moulds [23]. FT-NIR could hence also be a possible method to analyse yeast cell total fat content in a non-destructive way without the need for prior lipid extraction and the use of organic solvents.

The spectra produced by NIR spectroscopy contain a lot of information. It is sensitive to compound concentrations, physical structure, water content, etc. Since the NIR absorption bands are so broad and overlapping, the whole spectra or selected regions are selected for analysis. Multivariate analysis such as partial least squares (PLS) regression is often used to produce predictive models. A set of NIR calibration spectra are assigned to reference values obtained from available analytical methods [24]. The aim of this study was to establish a rapid non-destructive FT-NIR method for lipid quantification in oleaginous yeasts and additionally to minimise the usage of organic solvents used in conventional lipid concentration determination methods.

## Results

Pellets of yeast cells grown on different carbon sources at different cultivation times (see “Materials and methods”) were collected and analysed further. From the obtained spectra, a lipid prediction model was generated for *R. toruloides*.

For the FT-NIR calibration set, 60 unique samples of *R. toruloides* were analysed in triplicates, from which 179 spectra were used, followed by lipid extraction to provide a reference value. As an external validation set, 18 samples were collected and analysed in the same way. The FT-NIR wavenumber regions of interests for lipid concentration measurements were the following: 4167–4545 cm^−1^, 5600–6150 cm^−1^, 6900–7300 cm^−1^ and 8000–9000 cm^−1^. At these regions, C–H bonds are absorbing infrared light [25].

**Fig. 1:**
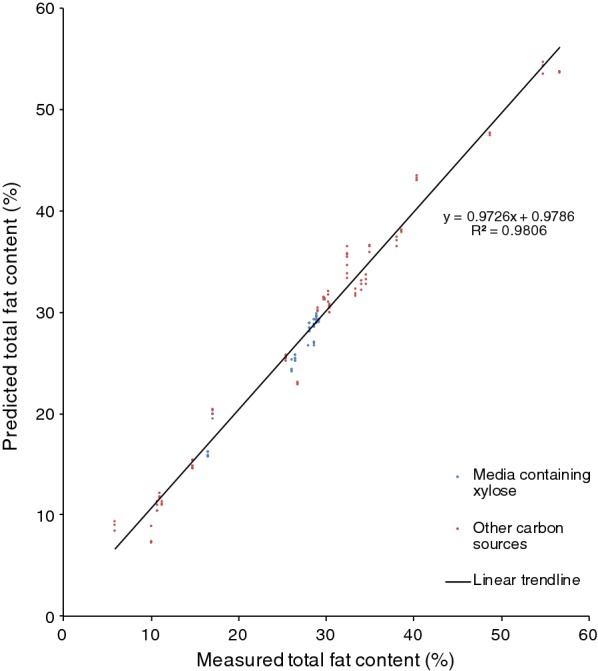
Cross-validation of the total fat content in *R. toruloides* CBS 14. From cultures grown in hemicellulose hydrolysate, mixed hydrolysate, crude glycerol or glucose with YNB. Blue dots represent cultures grown in media containing xylose as the main carbon source; red dots represent cultures grown in media containing other carbon sources (glycerol and glucose)

**Fig. 2:**
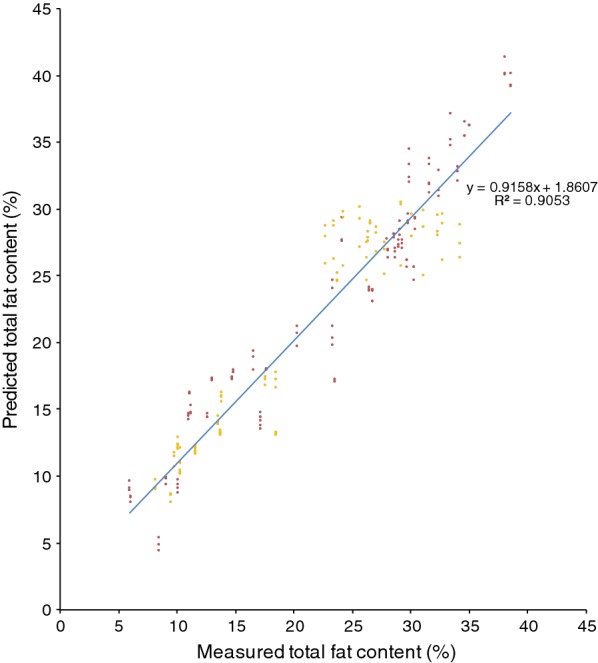
Cross-validation of the total fat content in the model of several combined yeast species (*R. toruloides*, *L. starkeyi*, *Y. lipolytica*), cultivations using hemicellulose hydrolysate, mixed hydrolysate, crude glycerol or glucose with YNB as cultivation media. Red dots represent red yeast; yellow dots represent *L. starkeyi* and *Y. lipolytica* samples

The determined fat content in the analysed *R. toruloides* samples varied between 5.9 and 59% of the cell dry weight (Figs. 1, 2).

**Fig. 3:**
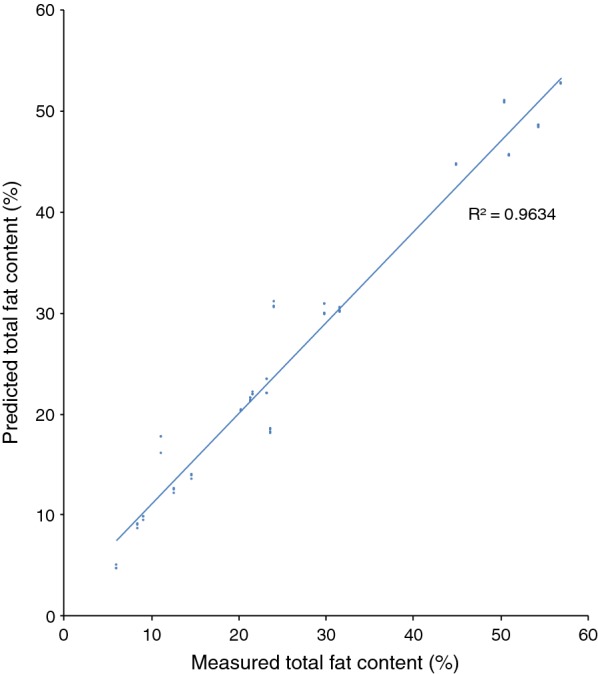
Test set validation of the total fat content in *R. toruloides* CBS 14 model. Cells were grown using YNB media with glucose, xylose and glycerol. A total 161 spectra were used for the calibration set of the model. Independent samples in form of 42 spectra were used as test set validation

**Fig. 4:**
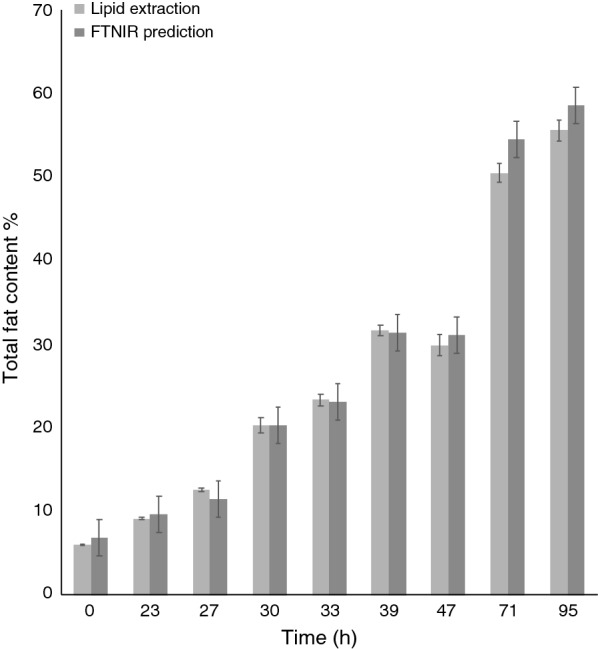
Comparison of *Rhodotorula* accuracy (dark grey bar) with traditional lipid extraction (light grey). Values were obtained by analysing test culture samples using the Quant2 analysis package. The samples were obtained from the cultivation of *R. toruloides* cultivated in a lignocellulosic hydrolysate (*n* = 27). The error in prediction was 2.17 for the FT-NIR model. Average difference between the lipid extraction results and the FT-NIR predictions was 5.2%

**Fig. 5:**
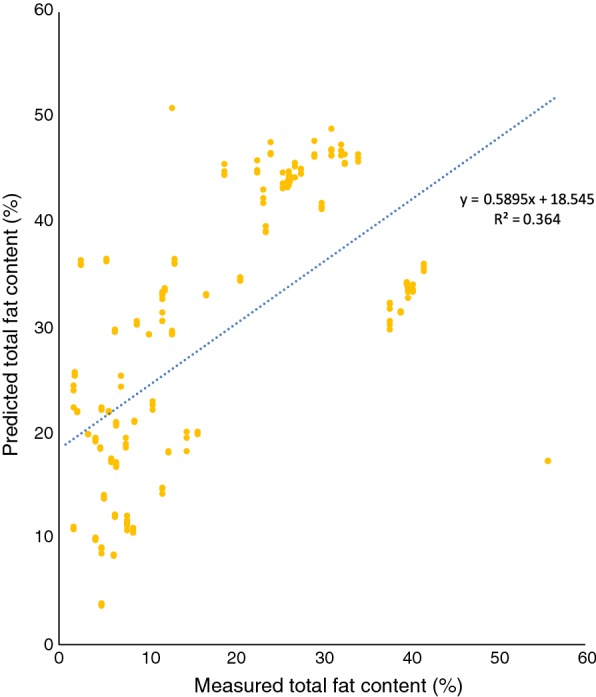
Using prediction model of *R. toruloides* on spectra of *L. starkeyi*, cultured in YNB with the addition of glycerol or glucose

An FT-NIR quantification model for *R. toruloides* total fat content was generated using the OPUS software included in the FT-NIR software package provided by the instrument manufacturer, and this FT-NIR quantification model was compared with the fat content data that were obtained from lipid extraction (Fig. 1). The calibration set produced for the *R. toruloides* cells with different lipid concentrations resulted in an FT-NIR quantification model with a cross-validation *R*^2^ value of 98% and a RMSECV value of 1.53 at rank 9. Vector normalisation was used for spectra pre-processing. The FT-NIR regions used to produce the model were: 8562.9–8038.4 cm^−1^ and 4485.9–4069.3 cm^−1^. The linear regression equation was: $y\, = \,0.9726x\, + \,0.9786$. Model accuracy was confirmed by comparing the lipid content of the samples predicted by the model with results obtained from the lipid extractions (Figs. 3 and 4). Additionally, separate test samples were used for external validation of the model showing that the produced model had an RMSEP value of 3.21. Samples were obtained from cultures grown in hemicellulose hydrolysate, mixed hydrolysate, crude glycerol or glucose with an addition of yeast nitrogen base (YNB). Figure 1 shows that the FT-NIR lipid predictions were not influenced by the medium composition. The yeast lipid accumulation data were compared with the prediction model data (see Fig. 4). In this comparison, the average difference between real data and predicted lipid content was 5.2%. To see whether the functioning *R. toruloides* model works on different yeast species, we tested the model on *Lipomyces starkeyi* samples, which resulted in large prediction errors (Fig. 5).

**Fig. 6:**
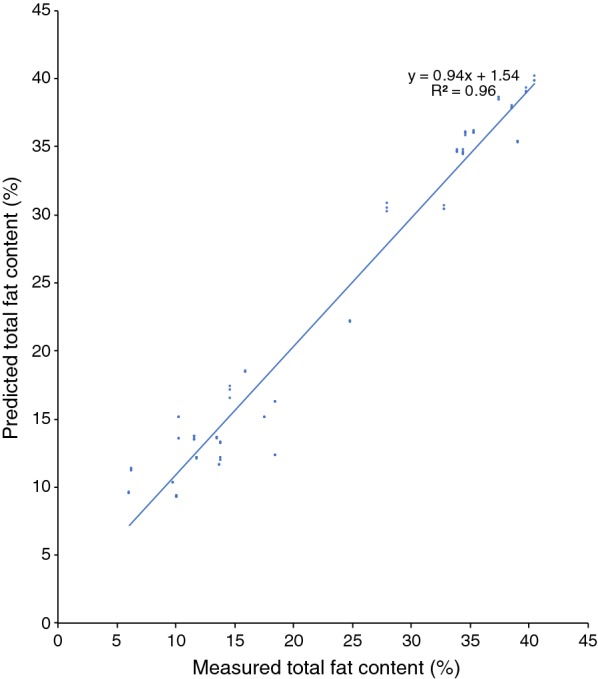
Cross-validation of the total fat content in the *Lipomyces* model of *L. starkeyi*, CBS 1807, cultivations using glycerol or glucose with YNB as cultivation media

**Fig. 7:**
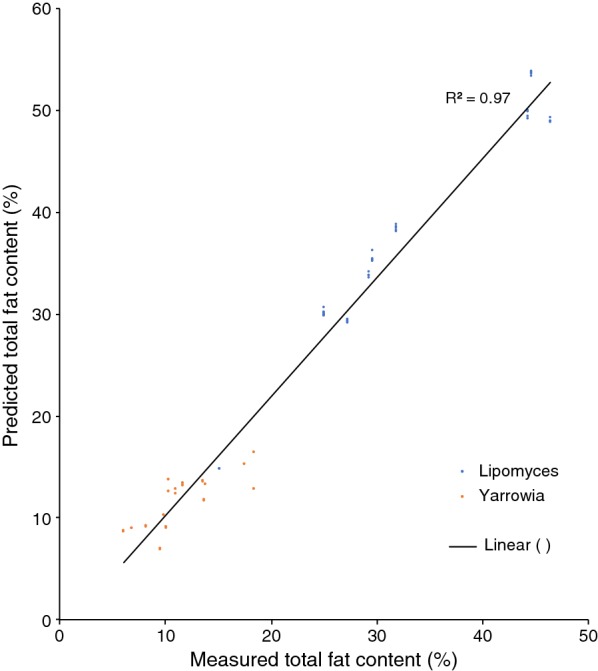
Test set validation of the total fat content in the *Lipomyces* model. Cells were grown using YNB media with glucose, xylose and glycerol. In total, 98 spectra from *L. starkeyi* and *Y. lipolytica* were used for the test set validation

In a similar fashion as for the model for *R. toruloides*, spectra and analytical data for *L. starkeyi* were used to create a *Lipomyces* lipid FT-NIR quantification model. The *Lipomyces* model consisted of 99 calibration spectra, with a *R*^2^ of 96% (Fig. 6) and a RMSECV of 2.4 at rank 2. Vector normalisation was used for spectra pre-processing, and FT-NIR regions used were: 8694.1–8061.5 cm^−1^, 7151.2–6649.8 cm^−1^ and 4466.6–3849.5 cm^−1^. The linear regression equation was: $y\, = \,0.94x\, + \,1.54$. To test the model, 98 spectra from *L. starkeyi* strains (CBS 1807, CBS 7544), and *Yarrowia lipolytica* CBS 6114 were used with a resulting validation RMSEP of 3.8 (Fig. 7).

Spectra and analytical data of *R. toruloides, L. starkeyi* and *Y. lipolytica* were used to create a combined lipid FT-NIR quantification model. The combined model consisted of 238 calibration spectra, with a *R*^2^ of 90.5% (Fig. 2) and a RMSECV of 2.76. The second derivative was used for spectra pre-processing, and FT-NIR regions used were: 8775.1–8034.5 cm^−1^ and 6001.8–5554.3 cm^−1^. The linear regression equation was: $y\, = \,0.9158x\, + \,1.8607$. To test the combined model, 55 spectra, including additionally *R. glutinis* CBS 2387 and *R. babjevae* DBVPG 8058, were used to test elasticity of the FT-NIR model with a resulting validation RMSEP of 3.22.

**Fig. 8:**
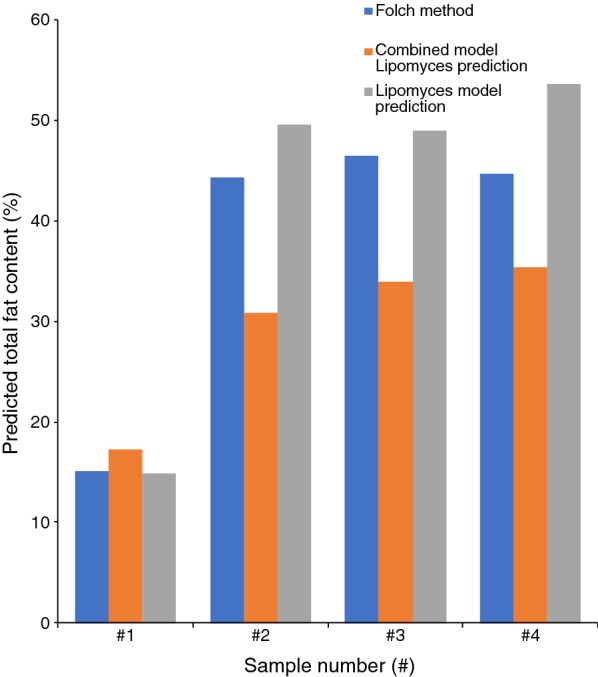
Comparison between combined model and *Lipomyces* model prediction to values obtained with Folch extraction on four samples of *Lipomyces starkeyi* CBS 1807

We compared predictions of lipid concentrations based on the combined model and the *Lipomyces* model with values determined by lipid extraction on four independent *L. starkeyi* CBS 1807 samples. The *Lipomyces* model values were more similar to the lipid extraction values than the combined model predictions (Fig. 8).

## Discussion

In the present study, we prove that it is possible to rapidly measure lipid content in red (*Rhodotorula* sp.) and other (*Lipomyces* *starkeyi*, *Yarrowia lipolytica*) yeast cells using FT-NIR spectroscopy with a high accuracy. It was only necessary to use organic solvent for lipid extractions during the establishment of the FT-NIR model, and afterwards they were only needed when clear outliers were present, or to further refine the FT-NIR model with new model data points. The verification of the produced FT-NIR lipid model on samples collected during cultivation of *R. toruloides* CBS 14 on different growth media, especially those taken during cultivation on lignocellulosic hydrolysate, further indicates that different cultivation methods do not substantially influence the FT-NIR-based lipid concentration predictions, as long as the analysed yeast cells are washed prior to the FT-NIR measurements. The FT-NIR model can also be used for analysing samples taken during cultivations using “difficult” cultivation media, to follow the lipid accumulation in the culture over time. In the previous studies, Laurens et al. established a lipid content model by using NIR measurements [21]. However, they only tested standard cultivation media, and used nitrogen limitation to get higher variation in lipid accumulation. In our study, we used different types of media, including lignocellulose hydrolysate, which, due to its complexity, could create problems if resulting in a variation in the lipid composition of the cells. In food science, it has been shown, for example, that it was possible to detect differences in the lipid composition due to variation of the cultivation conditions (e.g. region or temperature) [26, 27]. In contrast to this, our results clearly show that the cultivation media did not cause a significant variation in the lipid detection in yeast samples.

Laurens et al. [21] used a similar approach as in our study, but applied a different apparatus and processing of spectra. In particular, they used fatty acid methyl esters (FAME) instead of total fat. Unfortunately, both methods cannot directly be compared, as raw measuring data have to be processed by the according transformations.

It was not possible to use our existing red yeast model for the prediction of lipid content in non-pigmented yeasts. Similarly, the *Lipomyces* model could not be used for determining lipids in red yeasts. However, it was possible to use the *Lipomyces* model on *Y. lipolytica*. It needs to be tested on a per-case basis whether a strain belonging to another species can be analysed using a specific model. However, our results show that it is possible to combine species from different phyla of fungi when creating a model (Figs. 2, 8), in accordance with the results by Laurens et al. [21]. Compared to their model, our combined model was of acceptable quality. However, the model itself and the accuracy of it can be improved further by continuously adding additional samples [12, 25].

An advantage of using an FT-NIR-based lipid concentration determination method is that it is possible to share the model and data between different groups and users, thereby making it possible to expand the produced models by adding more samples from various sources. The data used to produce the prediction model are not restricted to one type of FT-NIR machine. This is already being done in, e.g., the bioethanol industry when measuring starch content in grains from different farms and harvest years, and at different production facilities. The models used by the industries are based on results from research activities on FT-NIR analysis of starch content in grains [28]. A future goal could be to establish an open access FT-NIR database used by academic groups carrying out research on lipid accumulating yeasts and industries focusing on yeast lipid production systems.

It has previously been pointed out and discussed that it can be difficult to create reliable and robust FT-NIR calibration models due to necessary knowledge of chemometrics [12]. In case of the OPUS software, which was used in our study, establishing and expanding models requires only basic knowledge and can be relatively rapidly performed. This simplifies the use of the model, and it is also possible to improve model accuracy by adding additional data points in the future. It should be noted that it is not required to use FT-NIR hardware for data processing (only for spectra collection), so the model itself can be analysed offline.

In the present study, the sample volumes used for classical lipid extraction and the measurement carried out by FT-NIR were approximately the same. However, it is possible to use smaller FT-NIR probes and thereby reduce the sample volume used [21, 29]. This is another advantage, especially when following lipid accumulation during the whole fermentation process when cell density is low, e.g. at the beginning of the cultivation, or during small-scale testing or screening.

We have created FT-NIR models to determine lipid content that is easy to implement for end user and that can be easily shared thanks to the possibility of exporting the model and sample values to other types of FT-NIR equipments with compatible software. As mentioned earlier, other studies tried different approaches with FT-IR and NIR for lipid detection [21, 22]. Both solutions are viable alternatives to our approach. With the FT-NIR method in combination with a dedicated software, a narrower spectra band than IR is used and most of the statistical operation is automated and performed without the user input. In all methods, freeze-dried sample is preferred, but wet yeast cells should be possible to be analysed, too, because there are examples of measuring fat in water solution like milk [20]. In the future, the technique could be also used to evaluate lipid classes and/or, fatty acid composition and level of oxidation in addition to total fat content similarly as described in [12, 21].

## Conclusions

To the best of our knowledge, we present for the first time working FT-NIR models for quantification of total fat content in oleaginous yeasts as fast and non-destructive alternatives for lipid extraction. These new models drastically reduce the time needed to obtain total fat content values, from few hours to a matter of minutes per sample analysed. Although gravimetric lipid determination is still more precise than prediction of the lipid content by the model, FT-NIR analyses will enable high-throughput measurements of lipid formation and kinetics studies of intracellular lipid accumulation. The precision of the model prediction can be increased further by including a growing number of new calibration values into the model.

## Materials and methods

### Yeast strains and cultivation conditions

The yeast strains used were *R. toruloides* CBS 14, *L. starkeyi* CBS 1807, *L. starkeyi* CBS 7544, *Y. lipolytica* CBS 6114, *R. glutinis* CBS 2387 (obtained from Centraalbureau voor Schimmelcultures, Utrecht, The Netherlands) and *R. babjevae* DBVPG 8058 (Industrial Yeast Collection, Perugia, Italy originally strain number J195, strain collection of the Department of Molecular Sciences, Swedish University of Agricultural Sciences, isolated from apple). They were stored at − 80 °C in 50% glycerol stock media. Before usage, the strains were transferred onto Yeast extract–Malt extract (YM) plates—10 g/L glucose (≥ 99%, Fluka Analytical, France), 5 g/L peptone (from casein, Merck KGaA, Germany), 3 g/L Yeast extract (Bacto™ Yeast Extract, BD, France), 3 g/L malt extract (Merck KGaA, Germany), 16 g/L agar (VWR Chemicals, Sweden). After 3 days of growth at 25 °C, the agar plates were stored at 4 °C. Pre-cultures were grown in YPD medium, in shake flasks at 25 °C for 72 h.

All media components except for yeast nitrogen base (YNB) and lignocellulose hydrolysates were autoclaved at 121 °C for 20 min. YNB and hydrolysate were sterile-filtered with a 0.2-µm filter (VWR International, LLC, USA).

All experimental media were based on YNB medium with additional salts: 1.7 g/L YNB (Difco™ yeast nitrogen base w/o Amino acids and Ammonium Sulphate, BD, France), 0.75 g/L Yeast extract (Bacto™ Yeast Extract, BD, France), 2 g/L (NH_4_)_2_HPO_4_ (≥ 98%, Sigma-Aldrich, USA), MgCL_2_ 1 g/L (Merck KGaA, Germany). The following carbon sources and additives were used: crude glycerol from biodiesel production (850 g/L glycerol (85% purity), provided by Perstorp AB), glucose (10–30 g/L), xylose (10–30 g/L), acetic acid (0.5–3 g/L, 95–97%, Sigma-Aldrich, USA), hemicellulose hydrolysate (from wheat straw—up to 40% v/v of hydrolysate used in experiments), cellulose hydrolysate (from wheat straw—up to 60% v/v of hydrolysate used in experiments). The hemicellulose/cellulose hydrolysate mixture carbon sources were determined by HPLC (Agilent 1100 Series, Germany) 50.8 g/L glucose, 29.3 g/L xylose and 5.2 g/L acetic acid. Wheat straw hydrolysate was obtained by steam explosion as described in Blomqvist et al. [2].

Yeast cultivations were performed in 2-L bioreactors (Minifors, Infors HT, Switzerland), 500 mL (Multifors, Infors HT, Switzerland) and in 100-mL baffled shake flasks (Werner-Glas, Sweden). The cultivations in bioreactors were performed with the following parameters: pH = 6 (acid 3 M H_3_PO_4_, base 5 M NaOH), DO = 21% O_2_, and temperature = 25 °C. Cultivations in shake flasks conditions: initial pH = 6, cultivation time 72 h, shaking at 130 rpm, 25 °C (Ecotron, Infors HT, Switzerland).

Each of the collected cultivation samples was washed with water two times and freeze-dried for 72 h in − 100 °C (CoolSafe Scanvac, LaboGene ApS, Denmark) to remove water and other possible compounds that can interfere with lipids in the spectra. After the collection of spectra, lipids from cells were extracted and quantified using a modified Folch method as described earlier [8].

A multi-purpose analyser (MPA) FT-NIR spectrometer from Bruker equipped with sample compartment and integrating sphere, combined with OPUS [OPUS ver. 7.5 build: 7.5.18 (20140810)], was used to obtain and analyse all FT-NIR spectra. All measurements were done in triplicates by using the integrated sphere compartment with the following parameters: Quartz beam splitter, RT_PbS detector, resolution of scan 16 cm^−1^, background measured internally. Glass vials containing freeze-dried cell pellets were scanned 32 times. Spectra were collected in the wavelength range from 4000 to 10,000 cm^−1^. A prediction model was generated with PLS regression using OPUS software Quant2 tool.

To ensure accuracy of the FT-NIR model, a smaller independent test set of samples, separate from calibration, were cultivated and analysed in the same manner. These samples were used to compare model prediction with actual extraction results.

**Fig. 9:**
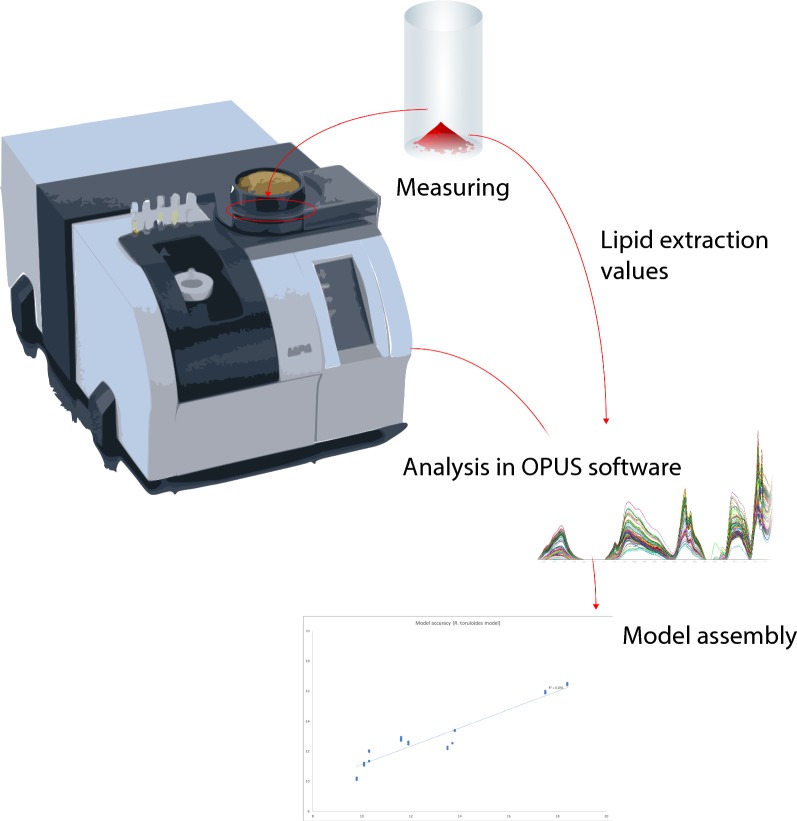
Simplified scheme of the model construction. In the first step, freeze-dried yeast cells are analysed by using MPA FT-NIR analyser. Next, lipid extraction is performed on the same cells and values are assigned to the respective spectra. Finally, when at least 50 samples are scanned and assigned to the analytical values, a model can be created with the help of Quant2 included in the OPUS software

The measuring procedure is illustrated in Fig. 9.

## Data Availability

The datasets used and/or analysed during the current study are available from the corresponding author on reasonable request.

## Abbreviations

IR: infrared
NIR: near infrared
FAME: fatty acid methyl esters
FT-IR: Fourier-transform infrared
FT-NIR: Fourier-transform near infrared
RMSECV: root mean square error of cross-validation
RMSEP: root mean square error of prediction
PLS: partial least square
MPA: multi-purpose analyser
YNB: yeast nitrogen base
YM: yeast extract–malt extract
YPD: yeast peptone dextrose
HIP: hexane isopropanol
